# 3D-Printed Polysaccharide Scaffolds with NIR-Triggered Activity for Diabetic Wound Healing

**DOI:** 10.1021/acsomega.5c05898

**Published:** 2025-09-11

**Authors:** Brianda M. Salazar Salas, Denis Scaini, Luis Fernando López Soto, Lucía Enríquez Rodríguez, Markel Lafuente-Merchan, Jorge Ordoyo-Pascual, Andya J. Ramírez-Irigoyen, José Luis Pedraz, Teresa del Castillo Castro

**Affiliations:** a Departamento de Investigación en Polímeros y Materiales, 27813 Universidad de Sonora, Hermosillo, Sonora 83000, Mexico; b NanoBioCel Research Group, Laboratory of Pharmacy and Pharmaceutical Technology, Department of Pharmacy and Food Science, Faculty of Pharmacy, University of the Basque Country (UPV/EHU), Vitoria-Gasteiz 01006, Spain; c Networking Research Center of Bioengineering, Biomaterials and Nanomedicine (CIBER-BBN), Institute of Health Carlos III, Vitoria-Gasteiz 28029, Spain; d NanoBioCel Research Group, Bioaraba, Vitoria-Gasteiz 01006, Spain; e Joint Research Laboratory (JRL) on Advanced Pharma Development, A Joint Venture of TECNALIA and University of the Basque Country, Centro de Investigación Lascaray ikergunea, Vitoria-Gasteiz 01006, Spain; f Departamento de Medicina y Ciencias de la Salud, 27813 Universidad de Sonora, Hermosillo, Sonora 83000, Mexico

## Abstract

Skin restoration in patients with diabetes constitutes a significant therapeutic challenge as sustained hyperglycemia interferes with fundamental processes such as angiogenesis, cell regeneration, and inflammation control. These alterations not only delay healing but also increase the risk of infections and complications. Emerging therapeutic strategies such as photothermal irradiation have gained attention for their potential to accelerate tissue repair. In this study, we developed novel three-dimensional (3D)-printed near-infrared (NIR)-responsive scaffolds based on chondroitin sulfate, hyaluronic acid, alginate, and nanofibrillated cellulose, with and without polydopamine photothermal nanoparticles, as a new approach to addressing complex tissue regeneration. The resulting 3D multicomponent scaffolds exhibited suitable morphology, swelling behavior, and biocompatibility for skin wound dressing. An *in vitro* scratch assay confirmed that the scaffold promotes keratinocyte migration and proliferation. *In vivo* studies demonstrated that treatment with an NIR-irradiated scaffold accelerated wound closure, leading to narrower scars and a denser dermis in diabetic rats. Notably, complete wound healing occurred 8 days earlier in animals treated with the nanocomposite scaffold under NIR irradiation compared to untreated controls. These findings highlight the therapeutic potential of multifunctional, NIR-responsive biomaterials and establish the proposed 3D-printed nanocomposite scaffold as a promising and innovative platform enhancing skin regeneration in challenging diabetic wound models.

## Graphical abstract

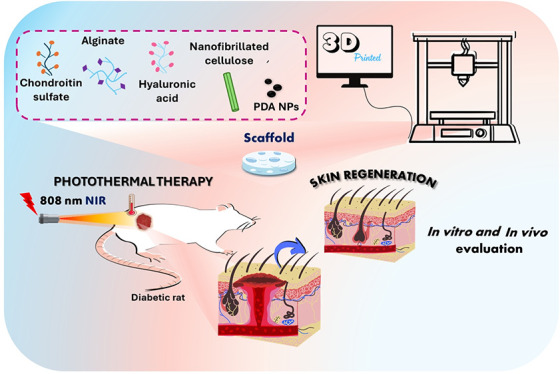

## 1 Introduction

The skin is one of the largest organs in vertebrates, serving essential protective, regulatory, and sensory functions. Damage or loss of skin integrity is a significant medical concern.^1^ Wound healing is a complex process that carries a constant risk of bacterial infections, especially in patients with chronic diseases such as diabetes or those in a poor healthcare environment (e.g., on the battlefield).^2^

According to the International Diabetes Federation (IDF) report, diabetes is a major global health problem, affecting 537 million adults aged 20–79 years. Approximately 25% of patients with diabetes develop diabetic foot ulcers, which frequently lead to lower limb amputations. This condition has significant socioeconomic implications; diabetes caused approximately 966 billion (USD) in total health-related expenditures globally and contributed to over 4 million deaths per year.^3^

Diabetic patients are severely affected by persistent inflammation, increasing their susceptibility to infectious processes.^4^ Serious bacterial infections can result in nonhealing wounds and even death.^2^ In this context, advanced therapeutic strategies such as near-infrared (NIR)-responsive scaffolds have emerged as promising approaches. Photothermal therapy (PTT) and photodynamic therapy (PDT), both light-activated tools, are being increasingly explored as noninvasive treatments to eradicate bacterial infections and enhance wound healing.^5–7^

Irradiation of materials containing photothermal agents can induce localized hyperthermia, effectively killing pathogens. Unlike antibiotic therapy, this approach may help prevent the development of drug resistance.^8^ NIR light offers deeper tissue penetration and higher spatial and temporal precision compared with visible light, thereby synergistically enhancing chronic wound healing. Specifically, NIR light in the 700–900 nm range can penetrate approximately 1–2 cm into soft tissues, which is sufficient to reach the dermis and underlying wound bed in most cutaneous applications.^9–11^

In this context, three-dimensional (3D)-printed scaffolds with photothermal capabilities have emerged as an innovative treatment option for patients with critical skin wounds.^12–14^ 3D printing enables the integration of various materials and bioactive ingredients, facilitating the design of conditions tailored and optimized for wound healing.^15–17^ This technique enables precise control over scaffold dimensions to match the wound area, ensuring a better conformity and contact with the tissue.

Photothermal scaffolds are typically prepared by encapsulating photothermal nanoparticles (NPs) within hydrophilic polymer networks. This approach combines the ability of photothermal agents to convert light energy into heat with the advantages of 3D polymer frameworks.^18^ Noble metal NPs and carbon-based nanomaterials have been the first-line option for PTT; however, these materials are not biodegradable and may pose toxicity concerns for clinical practice.^2^ In contrast, polydopamine (PDA) NPs, a conjugated polymer that mimics the naturally occurring melanin found in living organisms, have demonstrated excellent biocompatibility and photothermal performance, making them promising candidates for antibacterial therapies, particularly for wound infections commonly associated with diabetic conditions.^19,20^

In addition to technological advancements, there is an urgent need to find eco-sustainable, biodegradable polymers that can replace persistent materials in the 3D printing of skin regeneration, addressing the growing concerns about pollution associated with their disposal.^21^

In this study, we developed novel 3D-printed scaffolds via a green synthesis route, combining polysaccharides, including chondroitin sulfate (CS), hyaluronic acid (HA), sodium alginate (Alg), and nanofibrillated cellulose (NC), with and without PDA NPs as photothermal agent. In the context of skin regeneration, polysaccharides have demonstrated significant potential as scaffold-forming materials. CS has been shown to enhance dermal extracellular matrix remodeling, stimulate collagen synthesis, and support fibroblast activity.^22,23^ HA, a major glycosaminoglycan of the skin extracellular matrix, promotes fibroblast and keratinocyte migration and proliferation while maintaining a hydrated microenvironment essential for re-epithelialization.^24,25^ Alg, on the other hand, is widely used for creating skin-mimicking architectures due to its mild gelation conditions, ability to encapsulate cells, and capacity to support tissue repair.^26,27^ CN closely mimic the fibrous structure of native dermis, enhance the mechanical stability of scaffolds, and promote the adhesion and viability of skin cells.^28^

Although these individual biopolymers have been previously studied for wound healing, to the best of our knowledge, no previous work has reported a composite scaffold integrating all of these elements into a single system. The unique formulation presented in this study aims to synergize the biological and physicochemical advantages of each material while leveraging the photothermal capabilities of PDA under NIR irradiation to enhance skin regeneration.

## 2 Materials and Methods

### 2.1 Materials

Chondroitin sulfate (CS) (bovine, 100 000 Da, no. F-149110) and hyaluronic acid (HA) (no. F-077010) were obtained from Bioiberica (Barcelona, Spain). Ultrapure low-viscosity sodium alginate (Alg) with high guluronic acid content was purchased from FMC Biopolymer (Sandvika, Norway, no. BP-1806-13). A suspension of nanofibrillated cellulose (NC) was obtained from Sappi Europe (Brussels, Belgium, no. 271118N-BG12-48C). Dopamine hydrochloride (DA, CAS 62-31-7), Trizma base (99.9%, CAS 77-86-1), calcium chloride (CAS 10043-52-4), and 3-(4,5-dimethylthiazol-2-yl)­2,5-diphenyltetrazolium bromide (MTT) were purchased from Sigma-Aldrich. Fetal bovine serum (FBS) and penicillin/streptomycin (P/S) were acquired from Gibco (San Diego, USA). DPBS was purchased from Lonza (Porriño, Spain). Alamar blue was obtained from Bio-Rad (Madrid, Spain). A LIVE/DEAD Viability/Cytotoxicity kit was acquired from Life Technologies (Madrid, Spain). A DMEM-high glucose medium was obtained from ATCC (Virginia, USA). Deionized water purified by a Milli-Q Organex system (Millipore) was used to prepare the aqueous solutions.

### 2.2 PDA Nanoparticles

For the synthesis of PDA NPs, a 1 mM DA solution was prepared in a 5:1 (v/v) cosolvent mixture of buffer and ethanol. To initiate the DA autopolymerization, the pH was adjusted to 8.5 by using 100 mM Trizma base buffer, and the reaction medium was stirred at 500 rpm for 24 h at 60 °C. PDA NPs were purified by dialysis against Milli-Q water for 72 h with water changes every 4 h. UV–vis monitoring was performed to ensure the complete removal of the unreacted dopamine. Finally, the NPs were dried by lyophilization.

Nanoparticle morphology was analyzed using cryo-transmission electron microscopy (Cryo-TEM) with a Talos F200i (FEI) instrument, operating at an accelerating voltage of 200 keV and employing both bright-field and low-dose imaging modes. The hydrodynamic size and zeta potential of PDA NPs were determined using a Nano-ZS Zetasizer system (Malvern Instruments, UK). The absorbance of NPs was measured in a PerkinElmer Lambda 20 UV–vis spectrophotometer, and the Fourier transform infrared spectroscopy (FTIR) analysis was performed using the KBr technique on a PerkinElmer Frontier spectrometer.

### 2.3 Ink Formulations

The precursor ink of the CS-HA-Alg-NC scaffold was prepared as follows. CS and HA were dissolved at concentrations of 3 and 0.35% (w/v), respectively, in 1 mL of deionized water under stirring for 15 min at 37 °C. Alg (1%, w/v) was then added to the solution, and stirring continued for 2 h at 37 °C. Finally, 4 mL of the NC suspension (1.95%, w/v) was mixed with the polymer solution, and stirring was continued for an additional 1 h at 37 °C.

For the preparation of the PDA-containing ink, 2 mg of PDA NPs was dispersed in 1 mL of deionized water by shaking the suspension in a vortex Genius 3 (IKA, Alemania) for 5 min. The components CS, HA, Alg, and NC were used at the same concentration as in the PDA-free formulation. CS and HA were dissolved in the PDA NP suspension, and the same procedure described above was subsequently followed to obtain the precursor ink of CS-HA-Alg-NC_PDA_ (Figure 1a).

**Figure 1:**
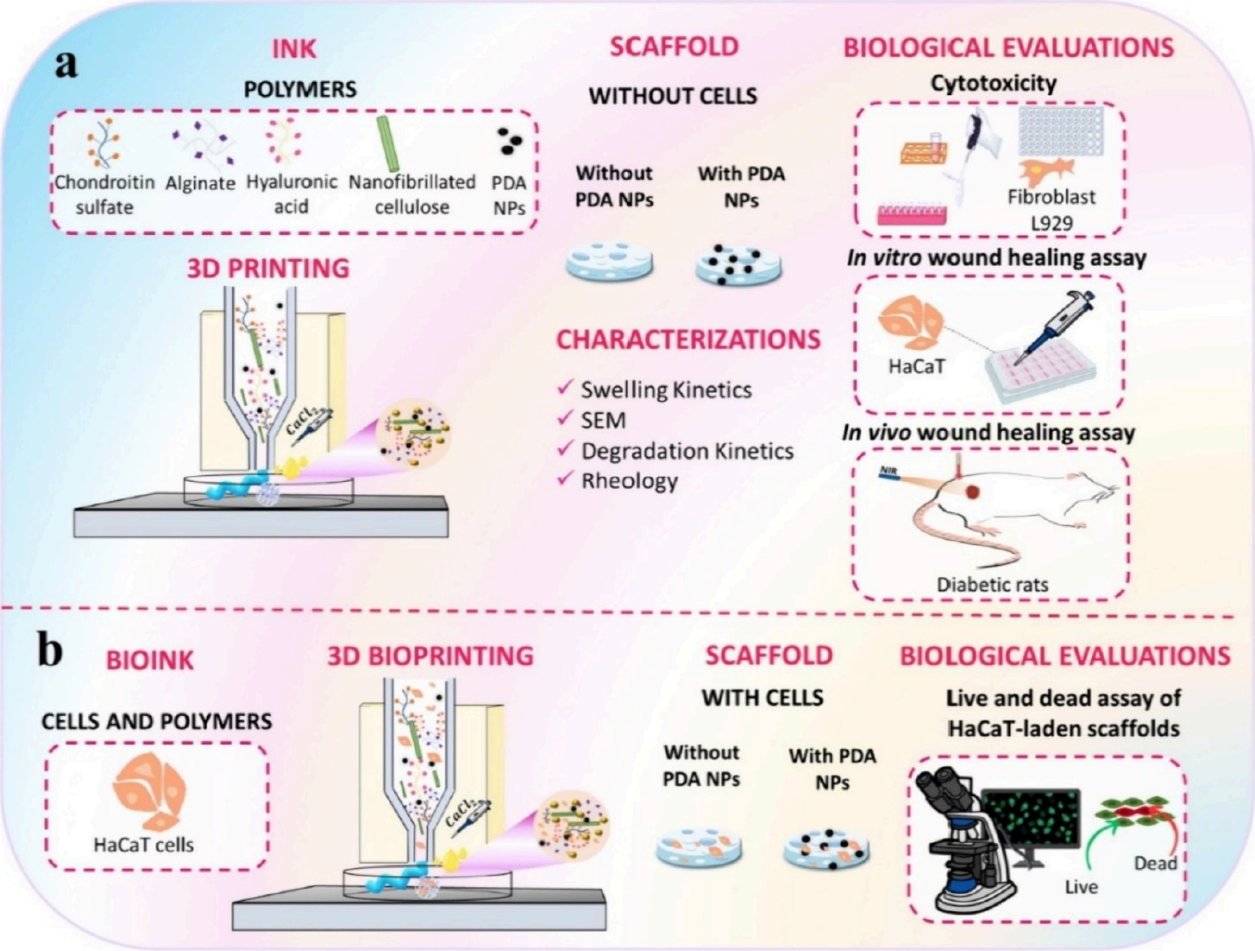
Schematic representation of the experimental protocols, illustrating the preparation of the 3D-printed scaffold (a) without cells and (b) with cells, along with their corresponding physicochemical and biological characterizations.

#### 2.3.1 Sterilization Process

Inks were sterilized using a short-cycle autoclaving procedure, which has been previously reported as a less harmful technique.^29^ This procedure was conducted by AJL Ophthalmic (Miñano, Spain) using an industrial autoclave model F0A2/B. The inks were initially subjected to temperatures of 15–18 °C and a pressure of 0.96 bar. For 22 min, the conditions were modified up to 123–124 °C and 3.60–3.70 bar. Then, sterilization occurred for 3.04 min. Subsequently, a cooling process took place for 26 min, decreasing the temperature and pressure to 50–55 °C and 1.60 bar, respectively. After 54 min, the autoclaving cycle was completed at 50 °C and 1.05 bar.

### 2.4 3D Printing of Scaffolds

Ink formulations with and without PDA NPs were transferred into 5 mL syringes and printed using a needle with a 0.51 mm diameter on a RegenHU bioprinter (R-GEN 100). Two-layer constructs with a diameter of 10 mm were printed in Petri dishes at plotting speed of 5 mm s^–1^ and extrusion pressure in the range of 20–25 kPa. Immediately after printing, 2 mL of 100 mM CaCl_2_ solution was added for cross-linking. Then, the scaffolds were washed with deionized water to remove excess cross-linking ions and subsequently freeze-dried.

### 2.5 Characterization of 3D-Printed Scaffolds

#### 2.5.1 Scanning Electron Microscopy (SEM)

Morphological analysis of the scaffolds was performed using a scanning electron microscope (SEM) model Emitech k550x, operated at an acceleration voltage of 15 kV. The samples were placed on carbon tape prior to SEM examination. Pore size dimensions were measured by using ImageJ software. The manual mode was utilized to measure the average diameters of the pores. At least 80 pores were measured in eight SEM micrographs for each scaffold type.

#### 2.5.2 Swelling Study

Swelling properties were investigated using the gravimetric method at 37 °C. Freeze-dried samples of known weight (*w*_0_) were immersed in DMEM.^30^ At specific time points (*t*), the samples were removed from the swelling medium, blotted, weighed (*w*_*t*_), and returned to the same bath until a constant weight was reached. The swelling percentage at time *t* was calculated from the following equation:

$$\mathrm{swelling}\;(\%)=\frac{w_{t}-w_{0}}{w_{0}}\times 100$$

#### 2.5.3 Degradation Study

Degradation studies were conducted by incubating the scaffolds in DMEM at 37 °C and monitoring their dimensions over 2 weeks.^31^ The samples were removed from the medium at different time intervals, and the surface moisture was carefully eliminated before the diameter was measured with a vernier caliper. The degradation percent at time *t* was determined by the equation:

$$\mathrm{degradation}\;(\%)=\frac{A_{t}-A_{0}}{A_{0}}\times 100$$

The terms *A*_0_ and *A*_*t*_ were the circular area of scaffolds at time zero and *t*, respectively.

#### 2.5.4 Rheology Measurements of Scaffolds

The rheological behavior of the scaffolds was analyzed using an AR100 rheometer from TA Instruments at 25 °C, employing a parallel plate fixture. Freshly prepared samples of 10 mm diameter and a thickness of 2 mm were used for the measurements. Oscillatory frequency sweeps were conducted from 0.1 to 100 Hz at a fixed strain of 2%.

#### 2.5.5 Cell Cytotoxicity Assessment

The *in vitro* cytotoxicity assessment of scaffolds was performed using the direct contact method based on ISO 10993-5:2009. Mouse L929 fibroblasts were used in all of the experiments. Circular scaffolds were placed in 96-well plates, and 10,000 cells/well were added. After 24 and 120 h, cell cytotoxicity was assessed using the MTT assay kit, following the manufacturer’s protocols. Cells cultured in a medium without scaffolds were used as the positive control, while a medium without scaffolds and cells served as the negative control.

### 2.6 Bioink Formulation and 3D Bioprinting

To evaluate the potential of the formulations for skin tissue engineering, keratinocytes (HaCaT cells) from ATCC (Virginia, USA) were added to CS-HA-Alg-NC and CS-HA-Alg-NC_PDA_ bioinks at a concentration of 5 × 10^6^ cells mL^–1^ (Figure 1b). The bioinks were loaded into 5 mL syringes, and the keratinocyte-laden scaffolds were then printed under aseptic conditions in Petri dishes at a plotting speed of 5 mm s^–1^ and an extrusion pressure in a range of 20–25 kPa.

#### 2.6.1 Cell Proliferation Assay in HaCaT Laden Scaffolds

Keratinocyte viability was determined by using a live/dead kit. The cell-laden scaffolds were stained with a 0.1 μM Calcein AM working solution in DPBS for 40 min in the dark at room temperature. Subsequently, 0.8 μM ethidium bromide solution was added, and the samples were further incubated for 10 min at 37 °C. Finally, the samples were washed with DPBS and observed under a Nikon TMS optical microscope (Virginia, USA).

### 2.7 *In Vitro* Wound-Healing Assay

An *in vitro* wound-healing test was conducted to evaluate the ability of different formulations to promote wound closure.^32^ HaCaT cells were seeded in 24-well plates at a density of 5 × 10^6^ cells cm^–2^ and incubated for 24 h in DMEM to form a confluent monolayer. The medium was removed, and the confluent cells were removed by scraping the surface of the culture well with a 200 μL pipet tip. The “scratch wound” creates a bare, cell-free space over which the remaining culture can migrate and mimic healing. The scratch-damaged HaCaT cells were washed with PBS to remove any cell fragments before incubating with the extracts of CS-HA-Alg-NC and CS-HA-Alg-NC_PDA_ scaffolds (media that had been in contact with the scaffolds for 24 h), with DMEM used as a control. Cell migration was monitored using a 24-well plate microincubator Cytation 1 BioTek (Winooski, USA), and images were taken every 10 min over 72 h of incubation. Images were analyzed using Gen5 version 3 software (BioTek) to assess wound closure sequentially throughout the assay.

### 2.8 *In Vitro* Photothermal Measurements

Scaffolds were immersed in 500 μL of PBS (pH 7.4, 100 nM) in a well plate,^33^ and then, the samples were irradiated with an NIR light 808 nm laser (Opto Engine, model PSU-III.LED) for 10 min at a radiation power of 1 W cm^–2^. The temperature increment was recorded using a thermographic camera (FLIR E53, USA).

### 2.9 *In Vivo* Diabetic Wound-Healing Test

Male Wistar rats were used to evaluate the effect of scaffolds on diabetic wound healing *in vivo*. A total of 30 male diabetic rats were randomly divided into six groups.

All rats were kept under a 12 h light/dark cycle at a controlled temperature of 25 °C, with water and food provided *ad libitum*. The experimental protocol was approved by the University of Sonora Ethics Committee (CEI-UNISON 17/2023) and adhered to the Mexican standard for the management and use of animals (NOM-033-ZOO-1995). Diabetes was induced via an intraperitoneal injection of streptozotocin (STZ) at a dose of 60 mg kg^–1^.^34,35^ After 7 days, animals with blood glucose levels above 300 mg dL^–1^ were selected for testing.

Rats were anesthetized by intramuscular injections of a ketamine/xylazine mixture (90:10) at a dosage of 70 mg kg^–1^ + 8 mg kg^–1^ of animal body weight. Dorsal-lateral area of each rodent was depilated, and circular incisions of 8.9 mm diameter were made using a disposable and sterile Dermal Punch.

Six groups of *n* = 5 were used in these experiments. The conditions tested were (i) scaffold −, NIR radiation −, (ii) CS-HA-Alg-NC, NIR radiation −, (iii) CS-HA-Alg-NC_PDA_, NIR radiation −, (iv) scaffold −, NIR radiation +, (v) CS-HA-Alg-NC, NIR radiation +, and (vi) CS-HA-Alg-NC_PDA_, NIR radiation +.

For irradiation treatments, the wound area (with or without the scaffolds) was irradiated with an NIR laser (808 nm) for 10 min at a radiation power of 1 W cm^–2^. The temperature of the lesion zone was monitored using thermographic images captured by the thermographic camera. The evolution of the wound was monitored by measuring its dimensions with a calibrated electronic vernier until complete closure.

### 2.10 Histological Analysis

After the wound-healing tests were completed, skin tissues near the wound were collected for histological analysis. The obtained skin tissues were fixed in 10% formalin for 24 h. After removal from the fixative solution, the samples underwent paraffin block preparation according to standard tissue preparation methods (dehydration, clarification, and molding). Finally, the prepared slices were stained with H&E and analyzed under a light microscope DM IL Led (Leica, Mexico).

### 2.11 Statistical Analysis

Statistical analyses of the results from swelling and degradation measurements, as well as wound-healing tests, were performed using analysis of variance (ANOVA) with a completely randomized design and factorial arrangement in NCSS software 2023. The values were considered significantly different at *p* < 0.05.

## 3 Results and Discussion

### 3.1 PDA NPs

PDA NPs were prepared by the autoxidation method in alkaline solution. Cryo-TEM images revealed a quasi-spherical morphology for NPs (Figure 2a). The PDA NPs were easily dispersed in aqueous solution, exhibiting a hydrodynamic size of 124 ± 0.13 nm (polydispersity of 0.12 ± 0.008) (Figure 2b) and a negative zeta potential of −41 ± 0.46 mV (Figure 2c). This negative zeta potential was attributed to phenolic hydroxyl groups on their surface.^36^ The UV–vis spectrum of PDA NPs showed a decrease in the typical absorption of DA at 280 nm, along with the appearance of a broad absorption band from 200 to 500 nm associated with the formation of 5,6-dihydroxyindole units in the polymer (Figure 2d).^37^ The FTIR spectrum of the DA monomer displayed typical features (Figure 2e), including bands at 3400–3000 cm^–1^ (O–H and N–H stretching), 1615 cm^–1^ (aromatic C=C stretching), 1522 cm^–1^ (N–H bending), 1250 cm^–1^ (C–N stretching vibration), and 1174 cm^–1^ (C–O stretching vibration). This last peak disappears in the spectrum of the PDA NPs confirming polymer formation. These results are consistent with previous findings for PDA NPs.^38–40^

**Figure 2:**
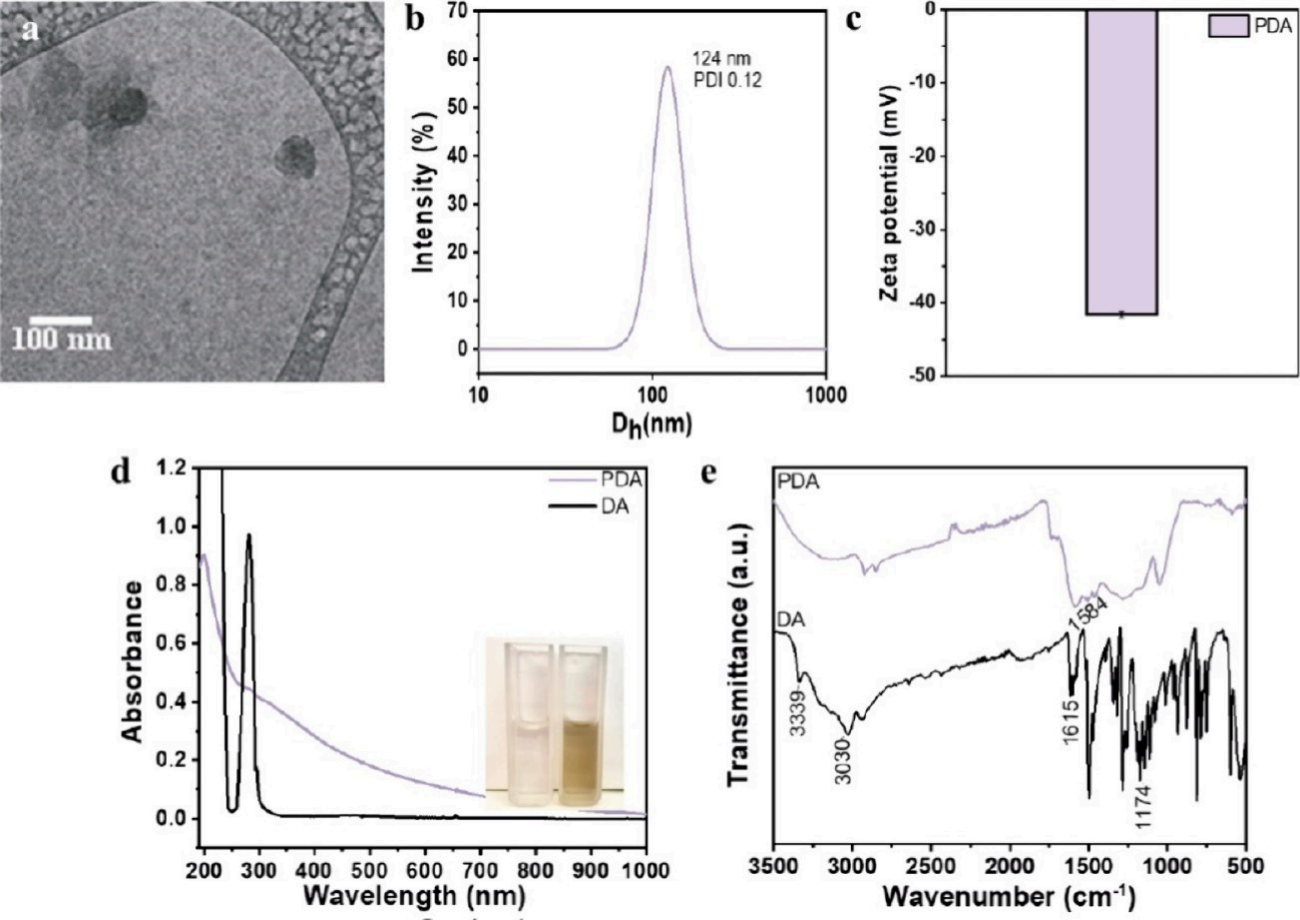
(a) Cryo-TEM image, (b) DLS distribution curve, and (c) zeta potential of PDA NPs. (d) Comparative UV–vis spectra (aqueous solutions) and (e) FTIR spectra of DA and PDA NPs.

### 3.2 Morphology of Scaffolds

Inks were extruded to produce 3D-printed scaffolds with uniform compositions and structures in both formulations. We selected a pressure range of 20–25 kPa, which provided the best balance between continuous flow and dimensional accuracy (Figure S1).

The incorporation of PDA NPs caused a visible color change in the scaffold from white to grayish brown. After the freeze-drying process, the 3D-printed scaffolds maintained their dimensions and geometry (Figure 3a,b). A porous microstructure is a highly desired feature of 3D-printed constructs intended for biomedical applications, as the interconnected voids promote water absorption, as well as the diffusion of nutrients, biomolecules, and cellular waste products, which are important events for tissue regeneration.^41^ Figures 3c and 3d show SEM micrographs of scaffold surfaces without and with PDA NPs, respectively. Both formulations exhibited a similar porous structure, with micrometer-sized pores randomly distributed across the material’s surface. The average pore size of the sample without PDA NPs was 83 ± 10 μm, while the scaffold containing PDA NPs had an average pore size of 62 ± 15 μm. The micrometer-sized pores of the scaffolds may contribute to enhanced cell adhesion, proliferation, and new tissue growth on the material.^42^

**Figure 3:**
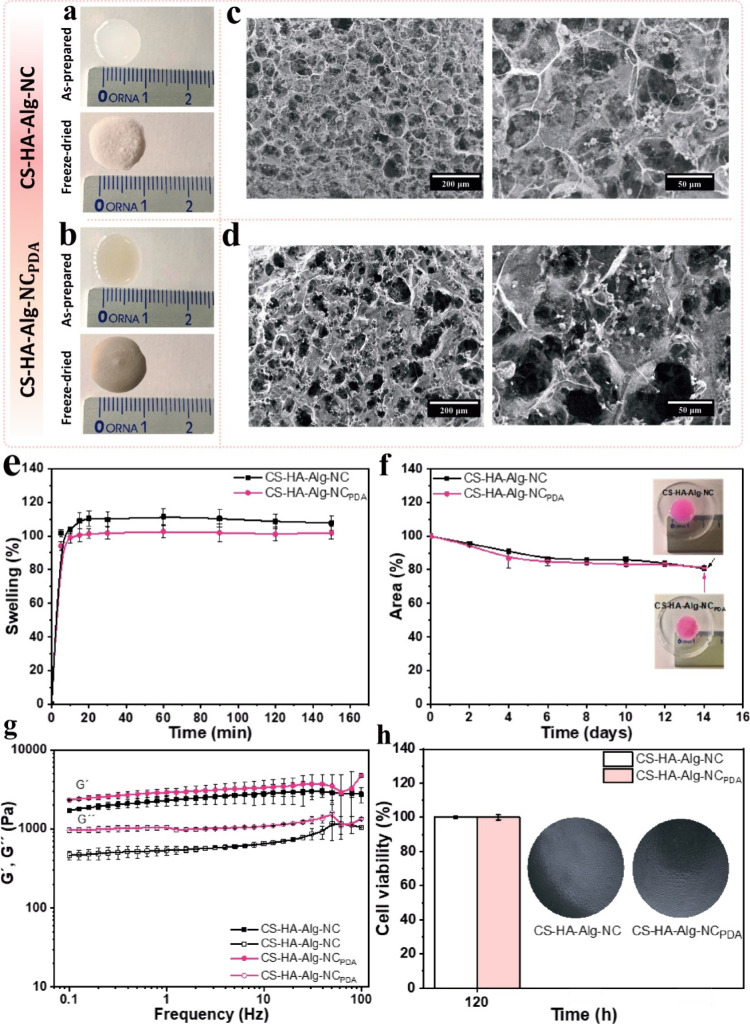
Images of fresh 3D-printed and freeze-drying scaffolds (a) without and (b) with PDA NPs. SEM micrographs of scaffolds (c) without and (d) with PDA NPs. (e) Swelling kinetics, (f) degradation profiles, and (g) rheology results of frequency sweeps for 3D-printed scaffolds. The storage modulus (*G*′) was represented by “open symbols” and the loss modulus (*G*″) by “closed symbols”. (h) MTT assay results. All data in the graphs are presented as mean values from three replicates, with error bars representing the standard deviation (±SD).

### 3.3 Swelling Capacity and Degradation Behavior

The degree of water absorption in scaffolds is a key factor to consider for biological applications. A high level of swelling enhances the scaffold’s capacity to load bioactive compounds or nutrients necessary for cell proliferation during incubation periods.^43^ Figure 3e shows the swelling kinetics of CS-HA-Alg-NC and CS-HA-Alg-NC_PDA_ scaffolds. Both samples swelled rapidly within the first 15 min of contact with DMEM at 37 °C, and no significant differences were observed in their equilibrium swelling levels. The swelling behavior of the scaffolds was associated with the water absorption capacity of their individual components and was consistent with the material’s microporous morphology.^44^

Figure 3f illustrates the degradation profiles of the scaffolds. Both samples exhibited a dimension reduction of approximately 20% after being immersed in DMEM for 14 days at 37 °C. For practical purposes, structurally stable scaffolds may be beneficial for cell adhesion and proliferation.^45^ In previous reports, printed scaffolds based on CS-chitosan degraded up to 80% in PBS at 37 °C for 21 days.^46^

### 3.4 Rheological Properties of Scaffolds

Figure 3g shows the frequency sweep profiles of the CS-HA-Alg-NC and CS-HA-Alg-NC_PDA_ scaffolds. The storage modulus (*G*′) and the loss modulus (*G*″) data revealed that both formulations exhibit predominantly solid-like behavior. Furthermore, minor variations in the dynamic moduli with frequency were observed across the entire frequency range, confirming the mechanical stability of the 3D-printed scaffolds. A slight increase in both *G*′ and *G*″ values was observed in the CS-HA-Alg-NC_PDA_ scaffold compared with the CS-HA-Alg-NC sample. This behavior may be due to PDA NPs, which likely restrict the mobility and flexibility of polymer chains within the matrix.^47,48^ A similar reinforcing effect has been observed for 3D-printed CS scaffolds containing gold nanorods.^47^

### 3.5 Cell Cytotoxicity Assessment

To evaluate the biocompatibility of 3D-printed scaffolds, direct contact tests of cytotoxicity were conducted using L929 cells, as shown in Figure 3h. Following 120 h of direct exposure to CS-HA-Alg-NC and CS-HA-Alg-NC_PDA_ scaffolds, the cell viability was observed to be above 100%. According to ISO 10993-5 standard, scaffolds were classified as noncytotoxic, corroborating their potential of materials for biomedical applications. Previous research has shown that L929 fibroblasts exhibit viability above 100% after 72 h of contact with injectable CS/poly­(γ-glutamic acid) hydrogels by using MTT assays.^49^ In another study, cell viabilities higher than 95% were observed for bone marrow mesenchymal stem cells after exposure to CS/gelatin-based scaffolds for 7 days, using cell counting kit-8.^45^

### 3.6 Cell Proliferation Assay in HaCaT Laden Scaffolds

The potential of CS-HA-Alg-NC and CS-HA-Alg-NC_PDA_ inks to promote keratinocyte growth was evaluated by adding HaCaT cells to the inks prior to bioprinting the scaffolds. Figure 4 displays fluorescent microscopy images of live/dead staining of HaCaT cells inside the scaffolds, which determines cell viability based on esterase activity and plasma membrane integrity. Calcein AM dye interacts with living cells (green), while the ethidium homodimer-1 stains dead cells (red).^50,51^

**Figure 4:**
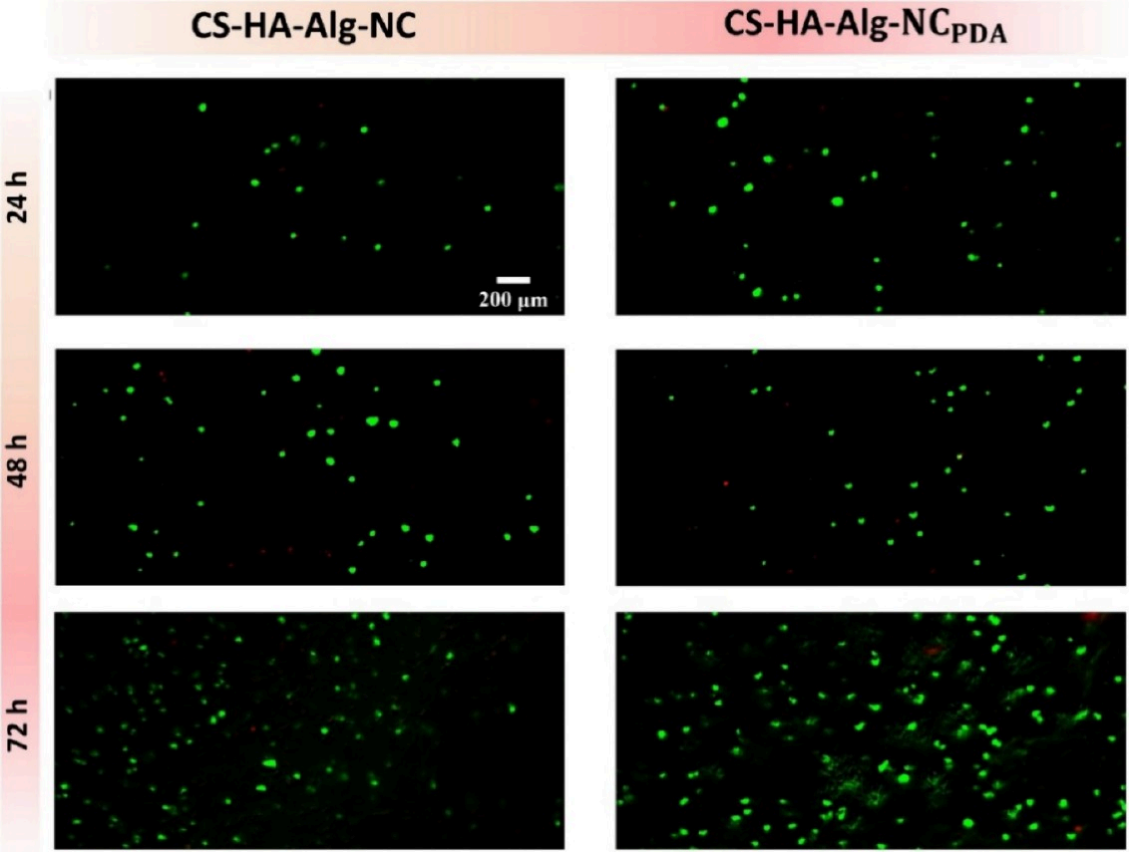
Live/dead cell staining images of HaCaT cells in bioprinted CS-HA-Alg-NC and CS-HA-Alg-NC_PDA_ scaffolds.

The results showed a higher percentage of live HaCaT cells compared to dead cells for both formulations, and a gradual increase in the cell number was observed over time. The porous morphology of both scaffolds may be beneficial for the nutrient supply and waste removal by encapsulated HaCaT cells. A previous report also evaluated the viability of HaCaT cells on HA/CS/poly­(vinyl alcohol) hydrogels using a live/dead staining assay, showing live HaCaT cells distributed within the scaffold matrix and their population increasing over time.^52^

### 3.7 *In Vitro* Wound-Healing Assay

Keratinocytes play a crucial role in the healing of skin wounds. Their rapid migration and proliferation at the wound site are essential for promoting re-epithelialization. 3D-printed scaffolds with appropriate viscoelastic properties can provide mechanical support for keratinocyte cells and also promote their proliferation rate.^53^ Figure 5a illustrates the *in vitro* wound-healing test performed with HaCaT cells in DMEM (control) and conditioned media containing extracts from CS-HA-Alg-NC and CS-HA-Alg-NC_PDA_ scaffolds. Figure 5b presents the wound closure data, while Figure 5c shows representative images of the scratch evolution over 72 h.

**Figure 5:**
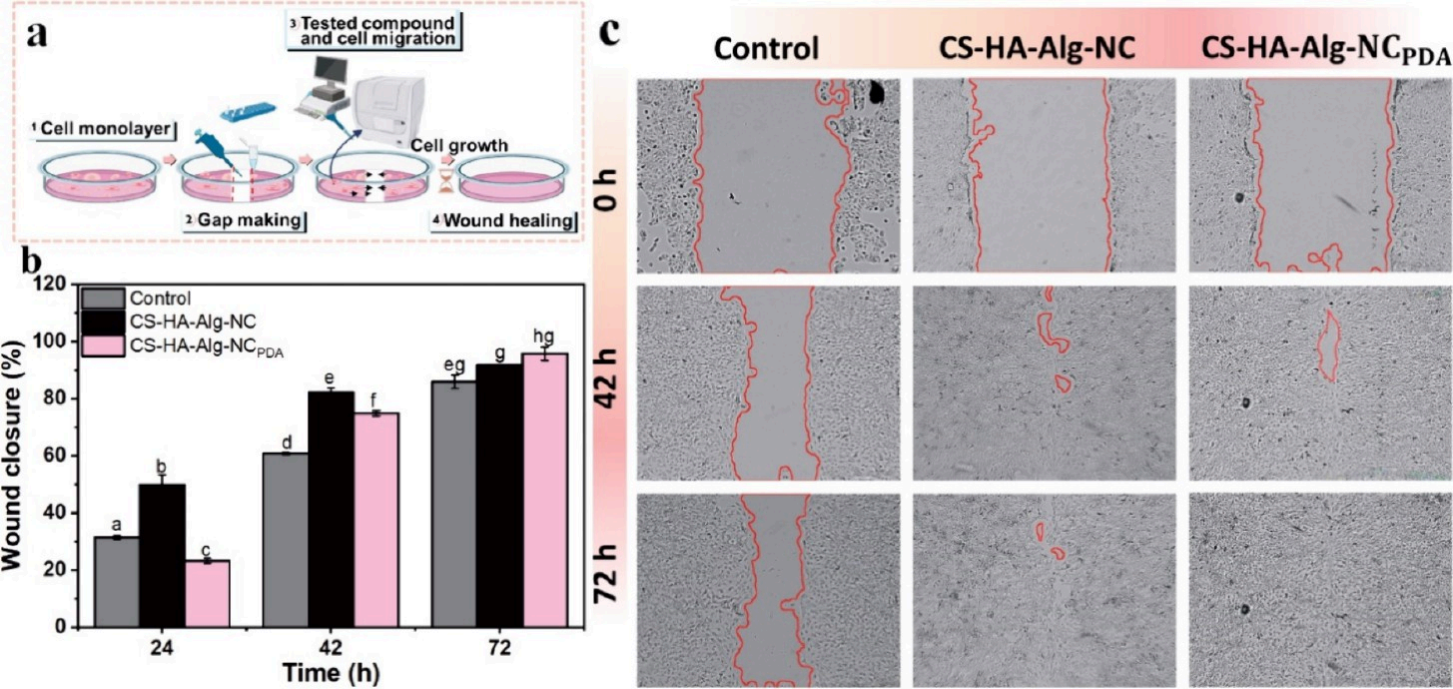
Effect of CS-HA-Alg-NC and CS-HA-Alg-NC_PDA_ extracts on the migration of HaCaT cells. (a) Sequence of the *in vitro* scratch wound-healing assay. (b) Quantification of the HaCaT migration rate, presented as mean values from three replicates, with error bars representing ± SD. Different letters (a–h) indicate significant differences (ANOVA, *p* < 0.05). (c) Representative optical images of keratinocyte migration in the scratch area.

Quantitative analysis of the migratory potential of keratinocytes with extracts confirmed the positive impact of the scaffolds. Both formulations accelerated cell migration and reduced the time required for gap closure. After 42 h, a significant increase in HaCaT cell proliferation was observed in the gaps treated with the scaffold extracts compared to the control. At 72 h postscratching, the scratch gap in the CS-HA-Alg-NC_PDA_-treated cells was completely closed. These results suggest that both formulations could support and promote the growth of epidermal keratinocytes. Previous work found 98% viability in HaCaT cells exposed to 42 μg mL^–1^ HA after 96 h.^54^ On the other hand, a higher percentage of HaCaT cell migration was obtained for scaffolds based on collagen/CS/PDA after 48 h, compared to the control.^55^

### 3.8 *In Vitro* Photothermal Measurements and *In Vivo* Diabetic Wound-Healing Assay

The thermal profiles of the PBS-hydrated CS-HA-Alg-NC and CS-HA-Alg-NC_PDA_ scaffolds were examined by exposing them to 808 nm of light for 10 min at a power of 1 W cm^–2^. Figure 6a shows the thermal profiles of the scaffolds as a function of irradiation time, along with the result of a control experiment using PBS. After 10 min of irradiation, the temperature of the PDA NPs-free scaffold and the PBS solution increased slightly from 25 to ∼30 °C, while the temperature of the CS-HA-Alg-NC_PDA_ sample increased its temperature to ∼55 °C. Literature reports indicate that a mild hyperthermia can reduce inflammation and facilitate angiogenesis and cell proliferation.^56^ These results confirm that the PDA NP-containing scaffold was able to convert NIR light into thermal energy due to the photothermal capacity of PDA NPs.

**Figure 6:**
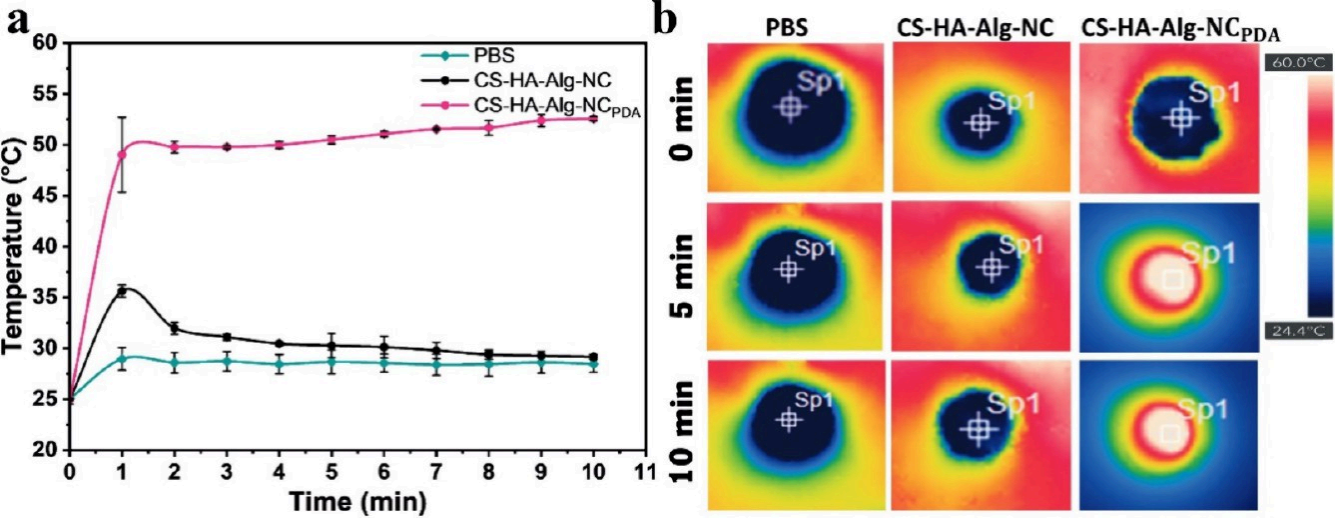
(a) *In vitro* temperature profiles of scaffolds and PBS solution under NIR laser irradiation (808 nm, 1 W cm^–2^). Data are presented as mean values from three replicates with error bars representing ± SD. (b) Representative thermographic images of different samples during *in vitro* NIR irradiation.

Thermographic images in Figure 6b illustrate the thermal evolution of the CS-HA-Alg-NC_PDA_ scaffold under NIR light exposure, in contrast to the nonresponsive behavior of samples without PDA.

Based on the satisfactory results of biological and physicochemical characterizations of the 3D-printed scaffolds, *in vivo* wound healing assays were performed on diabetic male rats. In the irradiation treatments, the wound area (with or without the scaffolds) was irradiated on days 0, 3, and 6 (Figure 7a). The PTT using PDA NPs operated in the NIR-I window (650–950 nm),^57^ which enables tissue penetration with minimal absorption by biological chromophores.^58,59^ Specifically, the 808 nm light used in this study can reach up to ∼10 mm, with optimal effectiveness below 5 mm, making it suitable for superficial to moderately deep wounds such as diabetic ulcers.^59–61^

**Figure 7:**
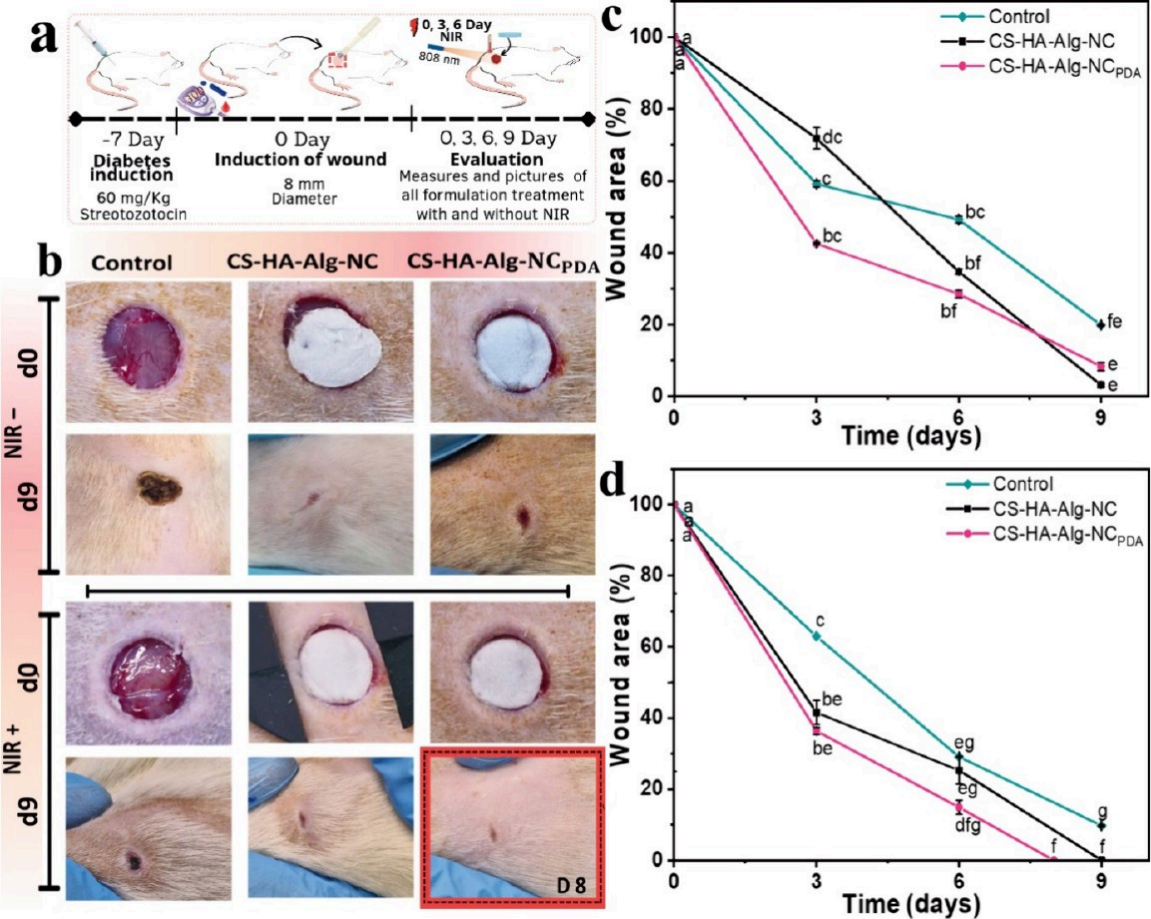
(a) Illustration of *in vivo* treatments in diabetic male Wistar rats, diabetes wounds were irradiated with an NIR laser (808 nm, 1 W cm^–2^) for 10 min, on days 0, 3, and 6 post injury. (b) Representative photographs of the wound areas for the different groups on days 0 and 9, except the red-labeled image that corresponds to day 8. Wound closure curves as a function of time for (c) nonirradiated groups and (d) NIR light-irradiated groups. Data are presented as mean values with error bars representing ± SD. Different letters indicate significant differences between groups (ANOVA, *p* < 0.05) (a–f).

Figure 7b presents images of the wound site for diabetic animals under different treatment conditions on days 0 and 9, except for the group treated with the nanocomposite scaffold and NIR light, for which images were taken on days 0 and 8. Figures 7c and 7d show the quantification of the wound area as a function of time for nonirradiated and irradiated diabetic rats, respectively.

The combined use of the CS-HA-Alg-NC scaffold and NIR irradiation significantly promoted the wound healing of skin lesions in diabetic rats. The wounds completely closed on day 9 in this group (CS-HA-Alg-NC, NIR radiation +), 3 days before the diabetic animals without irradiation (CS-HA-Alg-NC, NIR radiation −), and 7 days earlier than in the control group without scaffolds or NIR light (scaffold, NIR radiation −).

On the other hand, the diabetic wounds treated with the nanocomposite scaffold and irradiation (CS-HA-Alg-NC_PDA_, NIR radiation +) were completely healed on day 8 after injury, demonstrating a significantly faster healing process than in all other groups (Figure 7d). Wu et al. reported that diabetic wounds in rats treated with hydrogels based on PDA/acrylamide, MnO_2_ nanoparticles, and glucose oxidase healed completely by day 14, using NIR irradiation at 808 nm with a power of 1 W cm^–2^ for 10 min.^62^ Our findings indicated that the combined use of CS-HA-Alg-NC_PDA_ scaffolds and NIR photothermal therapy induces a synergistic effect on wound healing of skin lesions in diabetic rats. NIR irradiation combined with PDA-loaded hydrogels has shown a synergistic effect in diabetic wound treatment.^63^

Figure 8a shows representative thermographic images of the skin lesion region in different groups before and after 10 min of irradiation on days 0, 3, and 6. Figure 8b displays the temperature profiles of the wound site as a function of irradiation time at different treatment days. The temperature of wounds treated with CS-HA-Alg-NC_PDA_ increased rapidly to ∼40 °C in the first 2 min of irradiation and reached around 45 °C during treatment. On the other hand, the temperature increased to 34–38 °C in wounds treated with CS-HA-Alg-NC and remained within the range of 27–34 °C for the control group. This temperature trend was observed in the following days of NIR treatments, suggesting a sensitive relationship between temperature and wound-healing processes.

**Figure 8:**
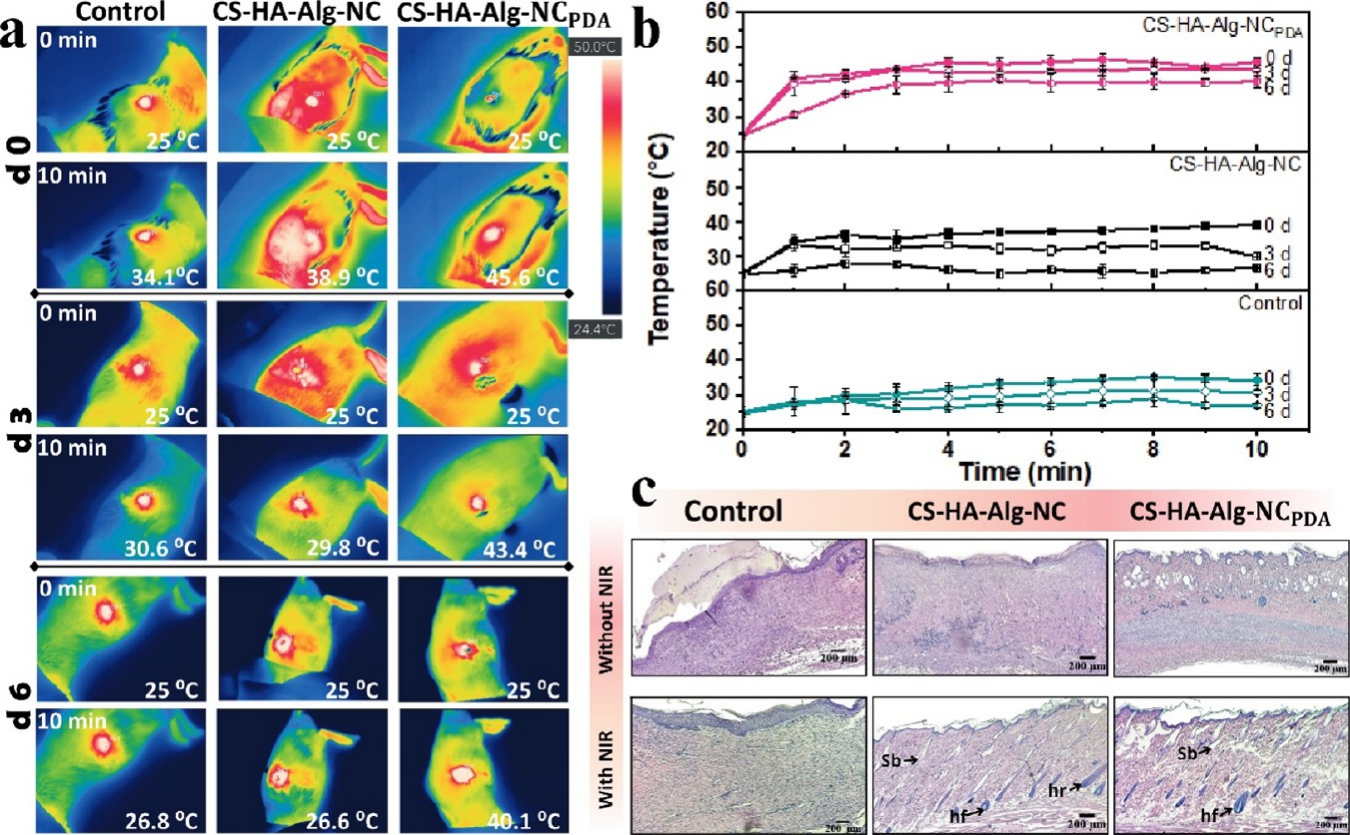
(a) Representative infrared thermographic images of *in vivo* treatments in diabetic male Wistar rats, wounds were irradiated with an NIR laser (808 nm, 1 W cm^–2^) for 10 min on days 0, 3, and 6 post injury. (b) Temperature profiles of wounds under NIR laser irradiation, data are presented as mean values with error bars representing ± SD. (c) Histological analysis of the wound-healing process. H&E staining was performed on wound tissue collected from the wound center, including both the scar and fully developed tissue, for nonirradiated groups and NIR-irradiated groups.

The temperature reached by the CS-HA-Alg-NC_PDA_ construct during *in vivo* assays (∼45 °C) was lower than the value obtained in the *in vitro* experiment (∼55 °C). This difference may be attributed to the heat dissipating effect of *in vivo* blood perfusion, as reported in studies by Chen et al. and Liu et al.^64,65^ Additionally, temperatures higher than 50 °C may cause cell death, as stated in the literature.^64^ *In vivo* wound-healing assays demonstrated the importance of tuning experimental parameters to optimize the beneficial effect of photothermal conversion on supporting wound healing.

### 3.9 Histological Evaluation

The wound-healing efficacy of the scaffolds was assessed through a histomorphological analysis. Compared to the control group, with and without irradiation the scaffold-treated groups exhibited thicker tissue formation (Figure 8c). Wounds treated with irradiated scaffolds (CS-HA-Alg-NC, NIR radiation + and CS-HA-Alg-NC_PDA_, NIR radiation + groups) showed faster formation and maturation of granulation tissue compared to the nonirradiated groups. Moreover, the wounds treated with irradiated scaffolds displayed narrower scars and a denser dermis with abundant sebaceous glands, hair follicles, and blood vessels, indicating accelerated recovery. In summary, the histological study confirmed the significant wound-healing potential of the designed scaffolds.

## 4 Conclusions

Novel multicomponent scaffolds with appropriate morphology, structural stability, swelling capacity, and biocompatibility were successfully prepared using 3D printing. These polysaccharide-based scaffolds provide an optimal architecture and create a conducive microenvironment for cell proliferation, closely mimicking natural tissue growth conditions. The inclusion of PDA NPs endowed the scaffolds with photothermal capabilities, enabling the efficient conversion of NIR light into heat. *In vitro* assays demonstrated the potential of these biomaterials to promote HaCaT cell proliferation. Furthermore, the synergistic combination of scaffold formulations and NIR irradiation significantly enhanced wound healing in skin lesions in diabetic rats. Real-time temperature monitoring during treatment highlighted the importance of fine-tuning the experimental parameters to optimize the therapeutic benefits of photothermal conversion. The unique composite scaffolds developed in this work are strong candidates for wound dressing. Additionally, 3D-printed scaffolds can serve as a valid, eco-friendly, and sustainable alternative to conventional nonbiodegradable photothermal materials.

## Supplementary Material



## Notes

(Figure S1) Design pattern for printability tests and optical microscope images of filaments printed at different pressures (PDF)
