# Supplementary material for: 3D-Printed Polysaccharide Scaffolds with NIR-Triggered Activity for Diabetic Wound Healing
Source: ACS Omega. 2025 Sep 11;10(37):43012–23. doi: 10.1021/acsomega.5c05898 (PMC12461302; doi:10.1021/acsomega.5c05898)
Supplement: Supplementary file 1 [file ao5c05898_si_001.pdf]

Supporting Information

3D-Printed Polysaccharide Scaffolds with NIR-Triggered Activity for Diabetic Wound Healing

*Brianda M. Salazar Salas [a], Denis Scaini [b, c, d, e], Luis Fernando López Soto [f], Lucía Enríquez Rodríguez [b, c, d, e], Markel Lafuente-Merchan [b, c, d, e], Jorge Ordoyo-Pascual [b, c, d, e], Andya J. Ramírez-Irigoyen [a], José Luis Pedraz [b, c, d, e,**], Teresa del Castillo Castro [a,*].*

[a] Departamento de Investigación en Polímeros y Materiales, Universidad de Sonora, 83000, Hermosillo, Sonora, Mexico
[b] NanoBioCel Research Group, Laboratory of Pharmacy and Pharmaceutical Technology, Department of Pharmacy and Food Science, Faculty of Pharmacy, University of the Basque Country (UPV/EHU), 01006, Vitoria-Gasteiz, Spain
[c] Networking Research Center of Bioengineering, Biomaterials and Nanomedicine (CIBER-BBN), Institute of Health Carlos III, Vitoria-Gasteiz, Spain
[d] Bioaraba, NanoBioCel Research Group, Vitoria-Gasteiz, Spain
[e] Joint Research Laboratory (JRL) on Advanced Pharma Development, A Joint Venture of TECNALIA and University of the Basque Country, Centro de Investigación Lascaray ikergunea, 01006 Vitoria-Gasteiz, Spain
[f] Departamento de Medicina y Ciencias de la Salud, Universidad de Sonora, 83000, Hermosillo, Sonora, Mexico

*Corresponding author

E-mail address: teresa.delcastillo@unison.mx *(Teresa del Castillo Castro)*

**Corresponding author

E-mail address: joseluis.pedraz@ehu.eus (José Luis Pedraz Muñoz)

Filament morphology and printability

To identify the shape-holding capacity of inks before crosslinking, a filament pattern was printed. Filament images were captured using the Nikon AZ100 microscope from Izasa Scientific (Barcelona, Spain) and analyzed using ImageJ software.

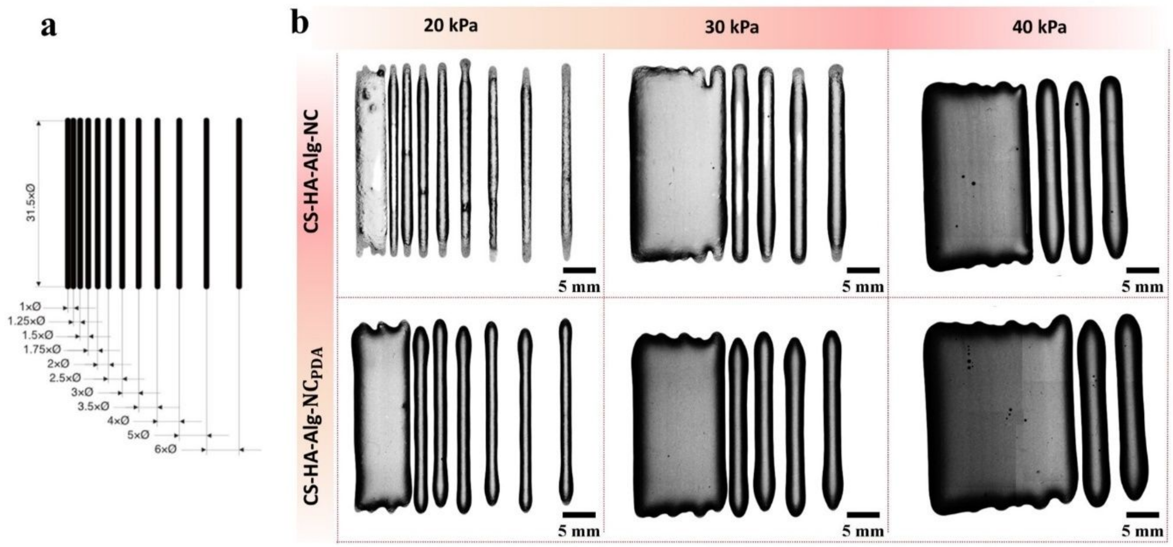

Figure. S1. (a) Design pattern for printability tests and (b) Optical microscope images of filaments printed at different pressures (20, 30, and 40 kPa).
